# An Exploration of the Serotonin System in Antisocial Boys with High Levels of Callous-Unemotional Traits

**DOI:** 10.1371/journal.pone.0056619

**Published:** 2013-02-15

**Authors:** Caroline Moul, Carol Dobson-Stone, John Brennan, David Hawes, Mark Dadds

**Affiliations:** 1 School of Psychology, University of New South Wales, Sydney, Australia; 2 Neuroscience Research Australia, Sydney, Australia; 3 School of Medical Sciences, University of New South Wales, Sydney, Australia; 4 Sydney Children's Hospital, Sydney, Australia; 5 School of Psychology, University of Sydney, Sydney, Australia; Yale University, United States of America

## Abstract

**Background:**

The serotonin system is thought to play a role in the aetiology of antisocial and aggressive behaviour in both adults and children however previous findings have been inconsistent. Recently, research has suggested that the function of the serotonin system may be specifically altered in a sub-set of antisocial populations – those with psychopathic (callous-unemotional) personality traits. We explored the relationships between callous-unemotional traits and functional polymorphisms of selected serotonin-system genes, and tested the association between callous-unemotional traits and serum serotonin levels independently of antisocial and aggressive behaviour.

**Method:**

Participants were boys with antisocial behaviour problems aged 3–16 years referred to University of New South Wales Child Behaviour Research Clinics. Participants volunteered either a blood or saliva sample from which levels of serum serotonin (*N* = 66) and/or serotonin-system single nucleotide polymorphisms (*N* = 157) were assayed.

**Results:**

Functional single nucleotide polymorphisms from the serotonin 1b receptor gene (*HTR1B*) and 2a receptor gene (*HTR2A*) were found to be associated with callous-unemotional traits. Serum serotonin level was a significant predictor of callous-unemotional traits; levels were significantly lower in boys with high callous-unemotional traits than in boys with low callous-unemotional traits.

**Conclusion:**

Results provide support to the emerging literature that argues for a genetically-driven system-wide alteration in serotonin function in the aetiology of callous-unemotional traits. The findings should be interpreted as preliminary and future research that aims to replicate and further investigate these results is required.

## Introduction

Of all the childhood psychopathologies, antisocial and aggressive behaviour problems such as Oppositional Defiant Disorder (ODD) and Conduct Disorder (CD) account for the greatest cost to psychological, psychiatric and social services [1]. ODD and CD include behaviours such as; spitefulness, arguing with adults, aggression towards others, destruction of property and violation of rules. The diagnoses identify heterogeneous groups; however, a characteristic that can be used to identify more homogenous groupings is that of callous-unemotional (CU) traits. These traits comprise low levels of guilt and shame, and a lack of empathy, and can be thought of as the downward extension of the affective and interpersonal characteristics of adult psychopathic personality traits [2]. Childhood antisocial behaviour problems characterised by high levels of CU traits have been found to be highly heritable [3], [4]. Viding et al. [4] found that genetic variability accounted for 71% of the variance in antisocial behaviour problems in 9 year old children with high levels of CU traits but only 36% in those with low levels of CU traits. High CU traits are also related to more severe and chronic antisocial behaviour [5], [6], greater use of proactive aggression [7], and specific patterns of neural dysfunction, specifically with regards to the amygdala [8]–[11]. Furthermore, people with high levels of CU traits (psychopathic personality) have been shown to have three specific cognitive and emotional deficits; a poor conditioned fear response, reduced ability to recognise fear, and deficits in stimulus-reinforcement tasks (see Moul et al. [12] for a review). These finding suggest that antisocial behaviour problems characterised by high levels of CU traits may have unique aetiological mechanisms associated with specific cognitive and affective impairments that are heavily dependent on genetics.

The Differential Amygdala Activation Model (DAAM) [12] is a recently developed model of amygdala function that has proposed a mechanism by which the subtle cognitive and emotional deficits characteristic of people with high CU traits, may develop. The DAAM posits that reduced serotonin neurotransmission may be integral to the pattern of amygdala activation it describes and hence to the development of the three deficits which, in turn, lead to high levels of CU traits. There is evidence to support the role of serotonin in the cognitive and emotional deficits characteristic of people with high levels of CU traits. Studies by Harmer et al. [13] and Attenburrow et al. [14] respectively found that acute administration of a serotonin selective reuptake inhibitor (SSRI) and ingestion of tryptophan (the natural precursor of serotonin) improved recognition of both fear and happiness. It has been shown that serotonin depletion in the prefrontal cortex of monkeys results in an impairment in stimulus-reinforcement learning [15]. Furthermore, low levels of serotonin in the basolateral amygdala are related to reductions in the conditioned fear response – a response which is reliably impaired in adults with high levels of psychopathic personality (CU traits) [16], [17].

Other researchers [18]–[21] have suggested that the serotonin system may be important in CU traits but we are aware of only two studies that have directly investigated the relationship between serotonergic function and CU traits. Fowler et al. (2009) found that a variable number tandem repeat (VNTR) polymorphism in the gene encoding monoamine oxidase A (responsible for catabolism of serotonin and other catecholamine neurotransmitters), and an insertion/deletion polymorphism in the *SLC6A4* promoter (5-HTTLPR) were associated with what the authors term the “emotional dysfunction” aspect of psychopathy (a construct that corresponds with CU traits) in a sample of adolescents with attention deficit hyperactivity disorder (ADHD) [18]. Sadeh et al. (2010) found that a gene-environment interaction between 5-HTTLPR and socioeconomic resources was associated with CU traits in adolescents. CU traits increased as SES decreased only among adolescents with the homozygous-long (l/l) genotype [22].

Serotonin has, however, been implicated in behaviours often found to be positively associated with CU traits; that is, aggression, antisocial behaviour and impulsivity. Genes encoding the serotonin 2a receptor (*HTR2A*) and tryptophan hydroxylase 1 (*TPH1*) have been associated with antisocial personality disorder (ASPD) in adult males [21]. Single nucleotide polymorphisms (SNPs) of the gene encoding the serotonin 1b receptor (*HTR1B*) have also been associated with anger and hostility [23]. A number of studies have used peripheral measures of serotonin and neuroendocrine challenge to demonstrate that the function of the serotonergic system may be altered in aggressive populations. For example, a negative relationship has been found between peripheral levels of serotonin (and its metabolite 5-HIAA) and antisocial/aggressive behaviours in both criminal and non-criminal males [24], [25]. Adults with heightened levels of aggression and antisocial behaviour have been shown to have a reduced reaction to fenfluramine challenge (a test of serotonin function in the hypothalamic-pituitary-adrenal axis) in comparison with healthy controls [26], [27]. In contrast Moffitt et al., [28] found that whole blood serotonin levels were positively associated with violence in adult males. In all, the vast majority of research using adult samples has demonstrated an inverse relationship between aggressive behaviour and serotonin system function (see [29] for a review).

The evidence to implicate low serotonergic function in childhood antisocial behaviour problems is less clear. Some studies have found a negative relationship between antisocial/aggressive behaviour and serotonin function [30], [31] that support the majority of findings from adult populations. Other studies, however, have found either no significant relationship [32], [33] or a positive relationship [34]–[37]. Interestingly, Halperin et al. [38] demonstrated that aggressive children with low-prolactin responses to fenfluramine challenge had significantly greater numbers of first- and second-degree relatives with aggressive and antisocial characteristics compared to both non-aggressive children and aggressive children with high-prolactin responses. The authors propose that these results suggest that there are two forms of aggression in children and only aggression that has its basis in familial biology is associated with diminished serotonin function. As commented by Manuck, Kaplan and Lotrich [29] this hypothesis is consistent with the identification by Moffitt [39] of different trajectories of childhood antisocial behaviour, one of which is described as “life-course-persistent” and thought to be strongly influenced by neurobiology. It is possible, therefore, as Manuck, Kaplan and Lotrich suggest, that it is this group of children with a familial, biological predisposition towards antisocial and aggressive behaviour that later comprise adult antisocial samples thereby offering a possible explanation for the general homogeneity of findings linking serotonin and aggression in adults.

It should be noted that heightened levels of aggression and antisocial behaviour do not demarcate high levels of CU traits. High levels of CU traits have, however, been shown to be a risk factor for the greater use of aggression and antisocial behaviours [5]–[7]. As such, it is possible that unmeasured, varying levels of CU traits within antisocial child samples may provide an explanation for the diversity of findings regarding the relationship between serotonin and antisocial and aggressive behaviours. Furthermore, the high heritability of CU traits and their association with more chronic and serious aggression and antisocial behaviour problems make them a strong candidate for the driving force behind the familial transmission of aggressive behaviour that Halperin et al. [38] argue is mediated, in part, by reduced central serotonin function.

In all, there is some indication that the function of the serotonin system may be important in CU traits. Current evidence, however, is inconsistent and the precise role of CU traits versus antisocial and aggressive behaviour, and other comorbidities, is unclear. Further, existing research has been limited to one or two functional polymorphisms at the expense of a more comprehensive assessment of the serotonin system.

This study explored the relationships between the serotonin system and CU traits in an antisocial sample with the general hypothesis that high levels of CU traits would be associated with indicators of reduced serotonin-system function. The majority of research into the role of serotonin has compared antisocial to non-antisocial groups and, as such, the unique association, if any, between CU traits and the serotonin system is unclear. As such, this research aimed to investigate the association between the serotonin system and CU traits within an entirely antisocial population. To this end, data regarding both serum serotonin levels and functional SNPs of serotonin-system genes were collected from a sample of boys with antisocial behaviour problems and varying levels of CU traits. It was predicted that boys with antisocial behaviour problems and high levels of CU traits would have lower levels of serum serotonin than boys with antisocial behaviour problems and low levels of CU traits. In addition, given the high heritability of antisocial behaviour problems in the presence of high levels of CU traits, it was hypothesized that CU traits would be significantly associated with functional polymorphisms of the serotonin-system. Given the exploratory nature of this research, 15 SNPs were selected from 7 genes with functions pertaining to the regulation of serotonin neurotransmission. SNPs were chosen on the basis of prior research regarding their functionality and association with psychopathologies. As the actual associations between SNPs and serotonin neurotransmission are dependent on factors other than just the functionality of the genotype there were no directional hypotheses made regarding the relationship between genotypes and CU traits. Two SNPs from the TPH1 gene were included even though their functionality is not yet clarified because this gene is involved in the regulation of serotonin synthesis in the body (as opposed to the brain) and given previous research to implicate low levels of peripheral serotonin this gene was an important inclusion. Details of the SNPs and their functionality are shown in table 1. The dominance of genotypes was inferred from previous research.

**Table 1:** Serotonin-System Gene Single Nucleotide Polymorphisms and their Functions.

Gene function	rs number	Major/minor (Inferred dominance)	Function of SNP	Associated with:	Influence of variant
*HTR1A* Codes for the 5-HT_1A_ receptor - regulation of serotonin neurotransmission	rs6295	C/G (G dominant)	Promoter polymorphism – blocks the function of repressors resulting in increased 5-HT_1A_ expression	Amygdala volume [65]; amygdala reactivity to threat [66]	G allele increases 5-HT_1A_ autoreceptor expression and reduces 5-HT release [66]
*HTR1B* Codes for presynaptic autoreceptor 5-HT_1B_ – inhibition of serotonin release	rs13212041	A/G (G dominant)	Attenuates microRNA mediated repression of gene expression	Conduct disorder [67]; variance in self-reported aggression and hostility [23]	AA genotypes have increased potential for the suppression of 5-HT_1B_ expression. [67]
	rs6296	G/C (C dominant)	Unknown – but in high LD with variants associated with transcriptional activity [63]	Aggressiveness and impulsivity [68]Aggression in children [69]	C allele has been associated with lower binding potential of 5HT_1B_ receptors in the brain but this is thought to be due to high LD with functional SNPs
	rs130058	A/T (T dominant)	Promoter polymorphism: in balance with rs11568817 regulates activity of 5-HT_1B_ [62].	Suicide and hostility in suicide completers [70]	A allele linked with higher transcriptional activity.
	rs11568817	T/G (Co-dominant)	Promoter polymorphism: in balance with rs130058 regulates activity of 5-HT_1B_	Alcohol dependence [71]	G allele linked with higher transcriptional activity [62]
*HTR2A* Codes for the 5-HT_2A_ receptor - regulation of serotonin neurotransmission	rs6314	C/T (T dominant)	Polymorphism results in a missense substitution at the 452^nd^ amino acid.	Antisocial behaviour and rule breaking in adolescents [61]	T allele results in amino acid substitution thought to result in changes to the secondary structure of the receptor [72]
	rs6311	C/T (T dominant)	Promoter polymorphism: involved in gene expression.	Aggression and impulsive behaviour [73]	T allele associated with increased 5-HT_2A_ receptor binding [74]
*HTR3B* Codes for the 5-HT_3B_ receptor – mediates fast excitatory serotonin transmission	rs1176744	T/G (G dominant)	Polymorphism results in a missense substitution which leads to an increased receptor response to 5-HT	Alcohol dependence [75]; receptor response to 5-HT [76]	G allele results in amino acid substitution which leads to increased receptor response to 5-HT
*TPH1* Codes for TPH - the rate limiting enzyme of serotonin synthesis external to the brain	rs1800532	C/A (Dominance unclear)	Function not yet clarified	Bipolar disorder and alcohol dependence [77]	Not yet clarified. A allele associated with blunted prolactin response to fenfluramine [78] and low levels of cerebrospinal fluid 5-Hydroxyindoleacetic acid [79]
	rs211105	T/G (Dominance unclear)	Function not yet clarified	Haplotype including rs211105 associated with schizophrenia [80]	Not yet clarified
*TPH2* Codes for the majority of TPH in the human brain [81]	rs4570625	G/T (T dominant)	Polymorphism involved in gene expression	Associated with major depressive disorder [82] amygdala reactivity to emotional stimuli increased in T allele carriers [83]	T allele thought to down-regulate gene expression [84]
	rs11178997	T/A (A dominant)	Polymorphism influences TPH2 transcriptional activity	Personality disorders [85]	A allele associated with reduced gene transcription [86]
	rs7305115	G/A (A dominant)	Polymorphism involved in gene expression	TPH2 expression [87]	A allele associated with greater gene expression [87]
	rs4565946	C/T (C dominant)	Function not yet clarified	Early-onset obsessive compulsive disorder [88]	Not yet clarified
*SLC6A4* Codes for 5HTT – responsible for the reuptake of serotonin into the presynaptic membrane.	rs2066713	C/T (T dominant)	Polymorphism associated with serum serotonin levels in males	Autism [89]	T allele associated with lower serum serotonin levels in males [90]

*Note*: 5-HT = 5 hydroxytryptamine (serotonin); TPH = tryptophan hydroxylase; 5HTT = serotonin transporter protein.

## Materials and Methods

### Ethics Statement

Ethics approval was from the Human Research Ethics Committee of the University of New South Wales (UNSW). Primary caregivers provided written informed consent to take part in the research and also provide written informed consent on behalf of their participating child/children. Adolescents (over the age of 12) were required to provide independent written informed consent. The donation of a blood/saliva sample was voluntary and participants and their parents were informed that choosing not to donate a biological sample would have no impact on their relationship with UNSW or their inclusion in the research program.

### Participants

All participants were referred to UNSW Child Behaviour Research Clinic (CBRC) or Royal Far West Children's Hospital (RFW), Sydney. Participants were a subset of a larger sample who met formal criteria for: 1) DSM-IV diagnosis [40] of antisocial behaviour problems (ODD or CD) using DISCAP structured interview [41]; 2) aged from 3–16 years for the genetic sample (*M* = 7.61, *SD* = 3.12) and aged 4–12 years for the serum sample (*M* = 6.89, *SD* = 2.25); 3) no major neurological/physical illness; 4) IQ>75; 5) have at least one set of measures of serotonin system SNPs or serum serotonin levels; 6) all known (at least 3) grandparents of Caucasian background (for participants included in the genetics sample); 7) provided written parental consent. Collection and analysis of DNA and serum levels evolved over several years and complete data were not available for all children; once all exclusions were in place, the sample sizes used for the main measures in the study were: serotonin system SNPs – Caucasian only (*N* = 157); serotonin serum levels – no ethnicity restrictions (*N* = 66); both genetic and serum serotonin level data – Caucasian only (*N* = 35). Boys using prescription drugs were included as it was predicted that their exclusion may have resulted in a biased sample. See table 2 for information regarding the types and usage of medications.

**Table 2:** The Percentage of Participants Using Medications in the Serum Serotonin Sample.

Medication type	Whole sample (N = 66)	Low CU (N = 47)	High CU (N = 19)
Psychostimulant	9.1	10.6	5.3
SSRI/MAOI	4.5	4.3	5.3
Antipsychotic	0.0	0.0	0.0
Bronchodilator	4.5	4.3	5.3
Other	3.0	4.3	0.0
Combinations#	3.0	2.1	5.3
Nil	75.8	74.5	78.9

*Note:*

# some participants were taking more than one type of medication from the list above. Some participants were taking antipsychotics but only in combination with other medications.

(SSRI = Selective Serotonin Reuptake Inhibitor, MAOI = Monoamine Oxidase Inhibitor).

### Measures

Parents completed the Antisocial Processes Screening Device (APSD) [42] and the Strengths and Difficulties Questionnaire [43]. These measures were chosen as they measure traits and behaviours associated with psychopathy and have been shown to be reliable in measuring these traits in child populations [43]–[46]. Family function was indexed via the Family Assessment Device-brief version (FAD) [47]. The FAD comprises a 12-item questionnaire that is based on the McMaster Model of Family Functioning and provides a measure of the structural and organizational properties of the family that have been found to distinguish between healthy and unhealthy families [47]–[49]. Current parental psychopathology was assessed via the short version of the Depression Anxiety Stress Scales (DASS) [50], [51]. The DASS is a self-report measure that indexes the emotional states of depression, anxiety and depression via three sub-scales of 7 items each. In addition, the parent also provided information regarding their home address. This information was used in conjunction with the Australian Bureau of Statistics (ABS) Socio-Economic Index of Area [52] to estimate the quality of the child's current surrounding environment. This scale provides a rating of the average standard of living for a given area on a ten-point scale with 1 indicating disadvantage and 10 indicating advantage.

CU traits were rated by parents using the UNSW system of combining items from the APSD [42] and prosocial scale of the SDQ [43]. This method has been validated by factor analysis [44] and has been used in previous research [44], [53], [54]. Cronbach's alpha coefficient for the UNSW CU traits scale in the sample of children that included both the genetic and the neurochemical samples was 0.81. Participants were divided into two groups for further analysis. Previous research suggests a range between the top 45% and 20% of aggressive/antisocial groups to represent high CU traits [6], [44], [55]; thus, boys with a CU traits score equal to or less than the 33^rd^ percentile were categorised as the low CU group. Boys with a CU traits score greater than this value were categorised into the high CU group.

Diagnoses were made using DSM-IV criteria by the assessing psychiatrist/psychologist using the DISCAP [41] with parents, and the child for those older than 8 years. Diagnoses were checked by having a second diagnostic team make an independent diagnosis. Kappa agreement across UNSW child mental health services on primary and secondary diagnoses were 0.772 and 0.770 respectively. Participants were rated by clinical psychologists, blind to levels of CU traits, on levels of antisocial behaviour problems, ADHD, autism spectrum disorders and anxiety and depression. Ratings were made on a scale of 0 to 6 with a rating of 4 or more indicating clinical levels of severity and a rating of 3 indicating borderline clinical severity. Boys with borderline ratings of antisocial behaviour problems were included in this study.

Adversity for the child was measured using the Quality of the Family Environment (QFE) [56], a clinician rating scale of the lowest quality of family environment to which the child was exposed during a substantial period (at least 1 year) before the age of 12. Ratings were made by a second naïve clinician on a subset of cases (*r* = 0.96). Sample characteristics are shown in table 3.

**Table 3:** Characteristics (mean (SD)) of the Genetic and Serum Samples.

	Genetics sample (*N* = 133)	Serum Sample (*N* = 66)
Age	7.56 (3.20)	6.89 (2.25)
QFE	74.28 (12.87)	74.69 (13.30)
ABS	7.80 (2.48)	8.61 (1.72)
Antisocial severity	4.05 (0.77)	4.06 (0.82)
ADHD severity	2.07 (2.02)	1.98 (2.09)
Anx/Dep severity	0.65 (1.37)	0.53 (1.28)
ASD severity	-	0.20 (0.81)
CU traits	7.26 (2.84)	7.50 (2.81)
CU traits 66^th^ percentile	8	8
Ethnicity	Caucasian 100%	Caucasian 66.66%
		Asian 16.66%
		Other/Unknown 16.66%

*Note*: QFE = Quality of the Family Environment, ABS = Australian Bureau of Statistics, Antisocial = Conduct Disorder/Oppositional Defiant Disorder, ADHD = Attention Deficit Hyperactivity Disorder, Anx/Dep = Anxiety/Depression, ASD = Autism Spectrum Disorder, CU = Callous-unemotional.

### Serum Serotonin Levels

66 participants gave blood samples between 8am and 10.45am (*M* = 9.03) at commercial pathology collection centres. 4 ml of blood was collected and left at room temperature for 30 minutes before spinning at 3000 rpm for 5–10 minutes. Serum was then poured off and kept frozen at −40 degrees centigrade until analysis. Serotonin levels were determined at the General Biochemistry Laboratory, Sydpath, St Vincent's Hospital, Sydney. Following solid phase extraction through Merck extrelut columns, serotonin in serum was measured by gas chromatography/mass spectrometry (GC/MS). This assay has a minimum detection level of 50 nmol/L and the assay is linear up to 7000 nmol/L. Intra-assay and inter-assay variation was 4% and 8.5%, respectively. All analyses were carried out blind to the participant's demographic information and diagnostic group. While relationships between peripheral blood concentrations and brain levels of serotonin are not fully understood, there is evidence that blood levels are a reliable marker of levels in the cerebral spinal fluid (CSF). Yan et al. [57] found large correlations between the concentration of serotonin in CSF and blood in 7 non-human primates; (e.g., at 9am *r* = 0.81). Furthermore, previous research has successfully documented significant relationships between aggression/antisocial behaviour and serotonin using peripheral blood measures [33], [37], [58].

### Serotonin-System Genotypes

122 participants chose to donate a saliva sample via Oragene saliva collection kits (http://www.dnagenotek.com/). DNA extraction rates were >95% for both blood and saliva methods. Genotypes were determined using iPLEX Gold™ primer extension followed by mass spectrometry analysis on the Sequenom MassARRAY system (Sequenom, San Diego, CA) by the Australian Genome Research Facility (AGRF: http://www.agrf.org.au/).

Serotonin-system SNPs were selected using dbSNP (www.ncbi.nlm.nih.gov/snp/). Genes that have been implicated in psychopathy (*HTR1B*, *HTR2A*, *TPH1*, *TPH2*) were prioritised so that at least two SNPs were selected for each of these genes. As this was an exploratory analysis of serotonin-system genetics it was important that the SNPs selected had known and relevant functionality. As such, with the exception of SNPs encoding TPH genes, all SNPs were functionally linked to serotonin-system gene expression by prior research (see table 1 for details). The SNPs were then reduced in number according to inclusion limitations of the Sequenom MassARRAY and the exclusion of SNPs in 100% linkage disequilibrium. The serotonin transporter promoter insertion/deletion polymorphism (5HTTLPR) has been implicated in psychopathy. However, due to funding and technical restraints, only SNP analyses were included in this research. The SNPs are shown in table 1.

### Statistical Analyses

Statistical significance levels for each comparison were adjusted to maintain *p* = 0.05 across multiple comparisons using False Discovery Rate (FDR) methods [59], [60]. All SNPs were in Hardy-Weinberg equilibrium with the exception of rs2066713 from the 5HTT gene (*p*<0.01). 12 samples were genotyped twice, once from saliva and once from blood, to provide a quality check. There was a perfect correlation of genotypes (*r* = 1.00, *p*<0.001) between samples which suggests that rs2066713 was not out of Hardy-Weinberg equilibrium due to genotyping error. This lack of Hardy-Weinberg equilibrium may be related to the fact that all children included in the analysis had antisocial behaviour problems and thus represent a stratified sample of the population.

The CU groups were first compared, by means of a univariate analysis of variance, on a number of possible covariates. These covariates were; age, antisocial behaviour severity, autism spectrum disorder (ASD) severity, attention deficit hyperactivity disorder (ADHD) severity, anxiety/depression severity and the Quality of the Family Environment (QFE) measure. The groups were found to differ significantly with regards to ASD severity (*F*(1,135) = 5.03, *p* = 0.03). The differences in all other variables between groups were found to be non-significant (*ps*>0.10). As such, children with ASD severity greater than zero (*N* = 24) were excluded from all further analyses leaving a sample of 133 boys.

Chi-square tests were conducted to compare frequencies of genotypes of all 15 SNPs between the high and low CU groups. As this was an exploratory analysis, all three genotypes (homozygous minor, heterozygous, homozygous major) were included in the Chi-square analyses where possible. For SNPs for which the expected distribution of genotypes violated the assumptions of Chi-square, genotypes were combined in accordance with knowledge regarding dominant/recessive alleles inferred from the existing literature (see table 1). In all cases, this meant combining minor homozygotes with heterozygotes to form “carrier” groups of the dominant allele.

Logistic regression was used to determine whether serum serotonin levels were a significant predictor of CU traits group after controlling for possible confounding variables. CU traits group was used as the dependent variable, covariates (age, time of blood collection, ABS Economic Index of Area, QFE, parental psychopathology (FAD and DASS scores), comorbid diagnosis severity) were entered in step 1 and serum serotonin level was entered in step 2.

## Results

### Relationship of CU traits to Serotonin-System Genotypes

Genotypes of *HTR1B* SNP rs11568817 (*χ^2^*(2, *N* = 133) = 11.63, *p* = 0.003) and *HTR2A* SNP rs6314 (*χ^2^* (1, *N* = 133) = 7.88, *p* = 0.005) were found to differ significantly in frequency between the high and low CU groups. Participants with high levels of CU traits were significantly more likely to be heterozygous (G/T) for SNP rs11568817 than participants low in CU traits. Participants homozygous for the major allele (CC) of SNP rs6314 were more likely to have high CU traits than heterozygotes (C/T) or minor homozygotes (TT). Results for all SNPs are shown in table 4. Haplotype analyses were conducted using PHASE software for SNPs from the 1b receptor gene, TPH1 and TPH2. The results of these analyses did not add to the results from the individual SNPs and, as such, are not described.

**Table 4:** Percentages and Chi-Square Statistic of Serotonin System Genotypes according to CU Group.

Gene	SNP	Low CU (*N* = 92)			High CU (*N* = 41)			χ^2^
		Minor Homozygote	Heterozygote	Major Homozygote	Minor Homozygote	Heterozygote	Major Homozygote	(*N* = 133)
***HTR1A***	rs6295	26	54	20	34	39	27	2.49
***HTR1B***	rs13212041	46		54	43		57	0.11
	rs6296	11	31	58	5	49	46	4.08
	rs130058	11	37	52	5	56	39	4.57
	rs11568817	22	37	41	7	68	24	11.63* #
***HTR2A***	rs6314	29		71	7		93	7.88* #
	rs6311	20	45	35	20	34	46	1.54
***HTR3B***	rs1176744	15	39	46	18	37	45	0.18
***TPH1***	rs1800532	12	38	50	20	44	37	2.49
	rs211105	6	47	47	10	37	53	1.18
***TPH2***	rs4570625	40		60	40		60	0.00
	rs11178997	10		90	15		85	0.72
	rs7305115	21	42	37	15	56	29	2.17
	rs4565946	19	52	29	13	52	35	0.64
***SLC6A4***	rs2066713	25	35	40	22	46	32	1.64

*Note*:

* represents p<0.01 (2-tailed),

# represents p<0.05 after FDR adjustment for 15 comparisons. SNPs located on the same chromosome are listed in order of their position along the gene.

### Relationship of CU traits to Serum Serotonin Levels

**Figure 1:**
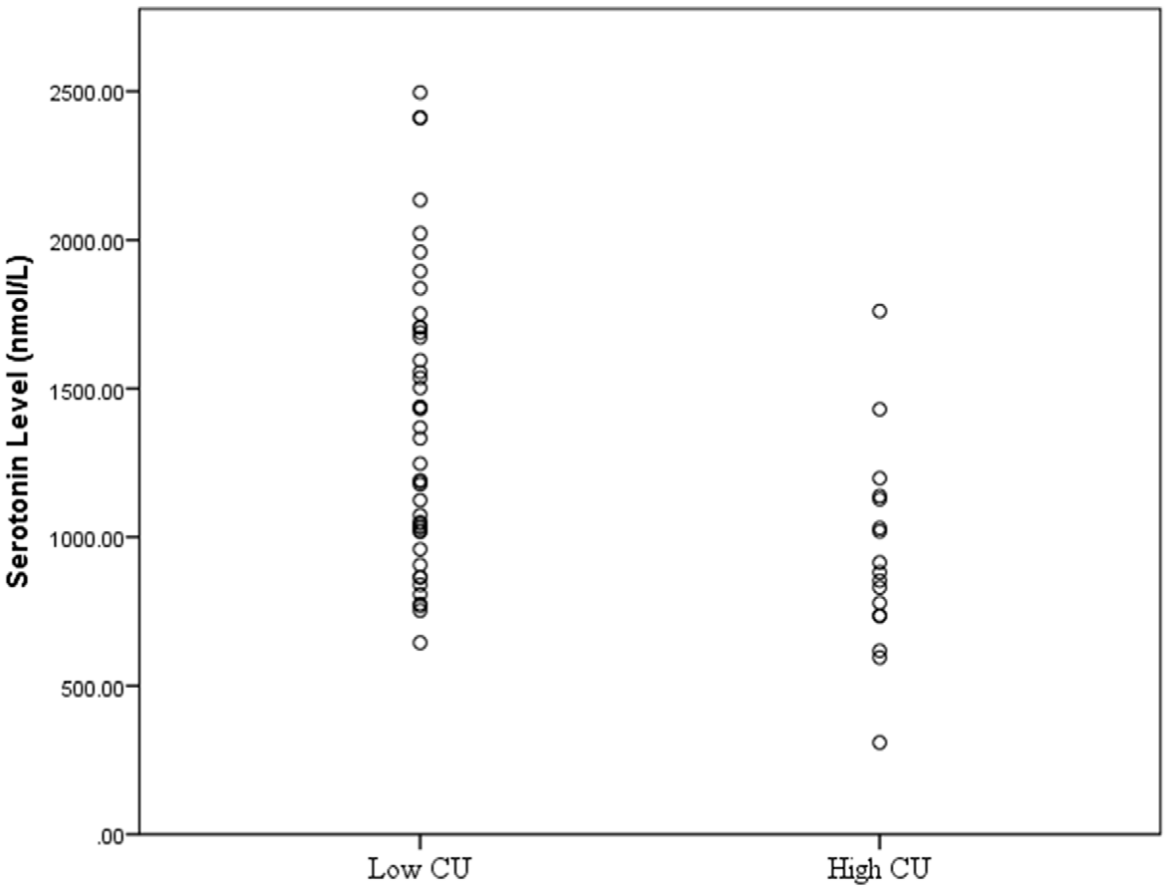
Distribution of serotonin levels in each of the CU trait groups.

The regression model at step 1 did not significantly predict CU group membership (χ*^2^* = 14.81, *df = 12*, *p* = 0.25). Of all the variables entered, only antisocial behaviour severity was a significant predictor of CU group membership (*β* = 1.15, Wald criteria = 5.05, *p* = 0.025, Exp (B) = 3.15). The inclusion of serotonin level at step 2 resulted in a significant regression model (χ*^2^* = 27.98, *df = 13*, *p* = 0.009). At step 2, serum serotonin level (*β* = −0.004, Wald criteria = 7.44, *p* = 0.006, Exp (B) = 0.996) was the only significant predictor of CU traits group. All other variables were non-significant (*p*>0.05). The low CU group (*N* = 47) had a mean serotonin level (*M* = 1324.9 nmol/L) that was significantly higher than that of the high CU group (*N* = 19) (*M* = 861.6 nmol/L). Figure 1 shows the distribution of serum serotonin levels in the high and low CU groups.

To be cautious and in order to be sure that the relationship between serotonin and CU traits was not being driven by medication effects, the regression analysis was repeated with boys using SSRIs/MAOIs (or a combination including SSRIs/MAOIs) (*N* = 5) excluded. Serum serotonin remained a significant predictor of CU traits group (*β* = −0.004, Wald criteria = 6.86, *p* = 0.009, Exp (B) = 0.996). None of the other variables were significant predictors of CU traits group when serotonin was included as a predictor.

### Genetics and Serotonin Levels

In order to explore the nature of the relationships between serotonin-system genetics and peripheral serotonin levels analyses of variance were conducted with serotonin level as the dependent variable and *HTR1B* rs11568817 and *HTR2A* rs6314 as the independent factors. It should be noted that sample sizes for these analyses were small; the number of participants for whom both serotonin level and genetic data were available (Caucasian only) and who were not taking SSRI/MAOIs was *N* = 35. As such, these data should be viewed as exploratory pilot results requiring replication in larger samples.

Serotonin level was not significantly correlated with any of the diagnostic variables, age or the Quality of the Family Environment measure in the sample (*ps*>0.20). As such, no variables were included as covariates in the analyses. rs11568817 was significantly associated with serum serotonin levels ((*F*(2,32) = 3.55, *p* = 0.04). Pairwise comparisons revealed that minor homozygotes had significantly lower mean levels of serum serotonin (*M* = 853.5, *SD* = 370) than major homozygotes (*M* = 1790.9, *SD* = 711.4, *p* = 0.037). There were, however, only 4 minor homozygotes and when the data from these participants were grouped with those from the heterozygotes the association between genotypes and serum serotonin levels was no longer significant (*F*(1,33) = 2.98, *p* = 0.09). rs6314 showed unequal variances between genotypes (Levene's test of equality of error variance (*F*(1,33) = 11.38, *p*<0.01)) and so a Kruskall-Wallis test was conducted. The test showed that rs6314 was not a significant predictor of serum serotonin levels (*H* = 0.36, *df* = 1, *p* = 0.55).

## Discussion

The results from analyses of both SNPs and peripheral serotonin levels suggest that differences in function of the serotonin system may be associated with high levels of CU traits in boys with antisocial/aggressive behaviours.

Two functional serotonin-system SNPs were significantly associated with CU traits. Genotypes of *HTR1B* rs11568817 were found to be significantly differently distributed in high versus low CU groups. Participants with high levels of CU traits were significantly more likely to be heterozygous (G/T) than participants low in CU traits. There thus appears to be a deleterious effect of the heterozygous genotype on CU traits: however, as true heterozygous effects are rare and the numbers of minor homozygotes in the high CU group are small, replication is required in larger cohorts to determine whether this is a true effect. Genotypes of *HTR2A* SNP rs6314 were also significantly differently distributed in high versus low CU groups. Participants homozygous for the major allele (CC) were more likely to have high CU traits than heterozygotes (C/T) or minor homozygotes (TT). The direction of this results is concordant with previous research that demonstrated that adolescents homozygous for the major (C) allele of rs6314 had significantly higher levels of antisocial behaviour and rule breaking, although notably not aggression, than heterozygotes and minor homozygotes [61].

The results from this exploratory study provide preliminary support to implicate the serotonin system in the aetiology of CU traits as proposed by the DAAM [12] and provide data to suggest that future research may benefit from comprehensive analyses of the serotonin 1b and 2a receptor genes.

This study also demonstrated that within a sample of children with antisocial behaviour problems there is a relationship between CU traits and peripheral serotonin levels that is independent from antisocial behaviour severity. It was found that serotonin level was a significant predictor of high CU traits even when antisocial behaviour severity was included as a covariate. This result supports the idea that previously reported mixed findings regarding the relationship between serotonin and antisocial and aggressive behaviour in children may be linked to variation in the levels of CU traits within and between samples.

With regards to the relationships between serotonin-system genetics and peripheral serotonin levels, the results provide tentative preliminary evidence to suggest that the G allele of *HTR1B* rs11568817 may be associated with lower peripheral serotonin levels. This analysis was, however, insufficiently powered and the results should be interpreted with appropriate caution. This direction of the association, however, is concordant with the idea that the minor allele of SNP rs11568817 engenders a risk for higher levels of CU traits, which in turn were found to be associated with lower levels of peripheral serotonin. Theoretically, this relationship between rs11568817 and peripheral serotonin levels is plausible. SNP rs11568817 is situated in the promoter region of the gene. Research has demonstrated that the minor allele of this SNP is associated with increased binding of transcription factors in the promoter region of the gene which produces a 2.3-fold increase in gene transcription [62]. As an autoreceptor, the serotonin 1b receptor helps to regulate the amount of serotonin released by the raphe nucleus. If gene transcription were increased, the density of 1b receptors would be amplified, which could lead to an increased inhibition of serotonin release. This is a possible mechanism by which the minor allele of SNP rs11568817 might lead to reduced peripheral serotonin levels and diminished serotonin-induced activation of the BLA as proposed by the DAAM [12]. It should be noted, however, that the effect of increased *HTR1B* gene expression on serotonin neurotransmission may vary depending on the location of the 5-HT1B receptor in the brain [63]. As such, any interpretation of the influence of changes in gene expression on serotonin neurotransmission should be made with caution.

There were a number of limitations to this research that require comment. First, this study was restricted by sample size. As the donation of a biological sample was a voluntary component of the overarching study the majority of donors chose to give a saliva sample from which peripheral levels of serotonin could not be obtained. As such, these results require replication with a larger sample for which both genetic data and peripheral serotonin levels would be necessary. In addition, power restrictions related to the sample size limited the number of SNPs that could be assessed. As such, while the results demonstrated that *HTR1B* and *HTR2B* warrant further investigation they do not rule out the possibility that genes for which few or only one SNP was tested may be important for CU traits. The research was also restricted by the use of a male-only sample. As such, no conclusions can be made about the relationship between serotonin-system function and CU traits in young girls with antisocial and aggressive behaviour. There is very limited research regarding the aetiology of CU traits in girls and this study requires replication in a female sample. The third limitation was that the genetic sample comprised children of Caucasian ethnicity only. While this was a necessary limitation in order to have a homogenous sample for genetic analyses it does, of course, mean that the results from this study cannot reliably be applied to children of other ethnic backgrounds. Ancestry was ascertained by self-report, rather than the more accurate method of genotyping ancestry-informative markers. However, it has been indicated that using self-reported ethnicity is an adequate substitute for ancestry markers in European ancestry populations, especially in studies examining a relatively small number of SNPs such as the current study [64]. Finally, it should be noted that the age range of children included in this research was large (3–16 years). Behavioural problems can present very differently across this age range and future research aimed at replicating these findings would benefit from testing restricted age ranges.

Future research should aim to replicate these findings in populations of adolescents and adults (both male and female). While CU traits in children are thought of by some as analogous to psychopathic personality traits in adults, the relationship between these traits and the function of neurochemical systems may not be the same in child and adult populations. The findings from this study may only apply to young male antisocial populations. Adolescence may significantly alter the associations between CU traits and the functions of the serotonin system. It is also likely that interaction effects of androgens may change the nature of associations between CU traits and neurochemical systems between genders. In order to comprehend the aetiology and development of CU traits it is necessary to get a better understanding of how these traits are characterised by the function of neurochemical systems in males and females across all developmental stages. It is also important for future research to go beyond exploratory association studies such as the one presented here and test specific hypotheses regarding functional SNPs and their impact on serotonin neurotransmission. We suggest that the *HTR1B* and *HTR2A* genes may be appropriate starting points.

In all, the results from this study suggest that the function of serotonin-system genes is important in the aetiology of CU traits. Functional SNPs from *HTR1B* and *HTR2A* were identified as significant predictors of CU traits in a sample of young boys with antisocial and aggressive behaviours. Future research should aim to replicate and further assess these results.
